# Inspiratory Muscle Training plus Pulmonary Rehabilitation versus Rehabilitation alone in COPD A Systematic Review of Randomized Controlled Trials

**DOI:** 10.12688/f1000research.175598.3

**Published:** 2026-05-05

**Authors:** Fahad Algharbi, Shibili Nuhmani, Mohammed Asiri, Alsayed Shanb, Mohammed Al-Subaiei

**Affiliations:** 1 Department of Physiotherapy, Imam Abdulrahman Bin Faisal University, Dammam, Eastern Province, Saudi Arabia; 2 Royal Commission Health Services program, Royal Commission for Jubail and Yanbu, Al Jubail, Eastern Province, Saudi Arabia; 3 Department of Respiratory Therapy, Maternity and Children Hospital, Kharj, Saudi Arabia

**Keywords:** Chronic Obstructive Pulmonary Disease, Pulmonary Rehabilitation, Respiratory training

## Abstract

**Background:**

Pulmonary rehabilitation (PR) is an established intervention for COPD, but the added value of inspiratory muscle training (IMT) within PR remains uncertain. This systematic review examined whether IMT plus PR provides benefits beyond PR alone in adults with moderate to severe COPD.

**Methods:**

PubMed, ScienceDirect, Cochrane Library, and Web of Science were searched from inception to January 2023. Randomized controlled trials (RCTs) comparing IMT+PR with PR alone in adults with moderate to severe COPD were included. Primary outcomes were inspiratory muscle strength (PImax), dyspnea, health related quality of life (HRQoL), exercise capacity [six minute walk test (6MWT)], and pulmonary function tests (PFTs). Risk of bias was assessed using the Cochrane RoB 2.0 tool.

**Results:**

Nine RCTs (n=295) met the inclusion criteria. IMT+PR improved PImax in 6/9 studies, with gains of 5.2 to 22.9 cmH _2_O. Dyspnea improved in 6/8 studies, often exceeding the minimal clinically important difference (MCID). HRQoL improved in all studies assessing this outcome (6/6), although superiority of IMT+PR over PR or control conditions was not consistently demonstrated. Exercise capacity findings were mixed, with significant within-group 6MWT gains in 4 of 7 studies but inconsistent between-group differences. PFTs (FEV _1_, FVC) were generally unchanged, while limited data from single-center trials suggest reductions in dynamic hyperinflation and small increases in inspiratory capacity.

**Conclusion:**

Adding IMT to PR meaningfully improves PImax and HRQoL in moderate to severe COPD, with frequent but less consistent benefits for dyspnea and 6MWT performance and minimal effect on spirometry. IMT may be most appropriate for patients with inspiratory muscle weakness (PImax <60 cmH _2_O or <50% predicted). Further RCTs should define optimal IMT protocols and clarify which COPD phenotypes derive the greatest benefit.

## Introduction

Chronic obstructive pulmonary disease (COPD) is a slowly progressive disorder characterized by persistent, largely irreversible airflow limitation resulting from a combination of small airway disease and parenchymal destruction (emphysema). COPD is a leading cause of morbidity and mortality worldwide, by 2030, it is projected to rank as the fifth leading cause of global disease burden (up from 12th in 1990) and the third leading cause of death (up from sixth in 1990), with an estimated three million deaths annually ( Mathers & Loncar, 2006). People with COPD typically present with chronic cough, sputum production, breathlessness, and wheezing, along with reduced exercise capacity and physical activity levels ( GBD, 2017). Dyspnea and fatigue are particularly prominent, and deconditioning frequently leads to further reductions in physical activity and exercise tolerance, thereby exacerbating functional limitations and disability. Multiple pathophysiological factors contribute to these impairments, including dynamic hyperinflation, gas exchange abnormalities, cardiovascular comorbidities, and respiratory muscle dysfunction.

Pulmonary rehabilitation (PR) is an evidence-based, multidisciplinary intervention that includes structured exercise training, education, psychosocial support, and nutritional counseling, and is recommended as a cornerstone of COPD management. PR has been shown to improve dyspnea, exercise capacity, physical fitness, and health-related quality of life, and to reduce hospital admissions and mortality, particularly in patients with frequent exacerbations ( Puhan et al., 2011). Inspiratory muscle training (IMT) has attracted particular interest as a potential adjunct to PR. The American Thoracic Society and European Respiratory Society recommend that IMT may be considered within PR programs for selected patients with COPD ( Spruit et al., 2013). IMT can be delivered using three main modalities (flow-resistive loading, volume-based devices, and pressure-threshold loading) with the primary aim of increasing inspiratory muscle strength and endurance, thereby improving overall functional capacity ( Geddes et al., 2005). The rationale for IMT in COPD is based on two main points: maximal inspiratory pressure (PImax) is frequently decreased, indicating weakened inspiratory muscles, and exercise capacity may be partly restricted due to fatigue of the respiratory muscles ( Charususin et al., 2013).

Core outcome measures of PR include respiratory muscle strength, six-minute walk distance (6MWD), pulmonary function indices, and dyspnea scales. IMT has been proposed as an adjunct to PR to further enhance these outcomes, yet its role remains controversial. Since its introduction in the 1980s, IMT has generated considerable debate owing to inconsistent findings regarding its clinical benefits in COPD. While the efficacy of IMT has been more clearly demonstrated in other populations including healthy individuals ( Illi et al., 2012), patients with neuromuscular disease ( Human and Morrow, 2021), chronic heart failure ( Wu et al., 2018), and asthma ( Lista-Paz et al., 2022) the largest body of evidence is associated with COPD. Within this population, IMT has been investigated both as a stand-alone intervention and in combination with other exercise modalities and/or comprehensive PR.

Numerous trials and meta-analyses have shown that IMT as a stand-alone therapy can improve inspiratory muscle strength, inspiratory endurance, functional exercise capacity, quality of life, and dyspnea. A systematic review and meta-analysis of 32 randomized controlled trials evaluating IMT alone in COPD demonstrated significant benefits over control for PImax (+13 cmH _2_O), inspiratory endurance time (+261 s), 6MWD (+32 m), quality of life (+3.8 points on the Chronic Respiratory Questionnaire), and dyspnea (−2.8 points on the Transitional Dyspnea Index) ( Gosselink et al., 2011). However, when IMT is integrated into a structured PR program, its additional contribution becomes less clear. Beaumont et al. (2018) synthesized 43 studies (642 patients) and reported that, although IMT improved inspiratory muscle strength, it did not confer additional benefits in dyspnea, quality of life, or exercise capacity when combined with PR. Similarly, the 2023 Cochrane review by Ammous et al. concluded that adding IMT to PR increased PImax by approximately 11.46 cmH _2_O but did not significantly enhance dyspnea or functional exercise performance compared with PR alone.

In contrast, individual high-quality randomized controlled trials have reported clinically important additive effects of IMT when combined with PR in carefully selected patients. Charususin et al. (2018), in a large multicenter trial (n = 219) of COPD patients with inspiratory muscle weakness (defined as PImax <60 cmH _2_O or <50% predicted), found that adding IMT to PR produced significantly greater improvements in endurance cycling time (+225 s vs. +163 s), endurance breathing time (+353 s vs. +162 s), and PImax (+22 cmH _2_O vs. +9 cmH _2_O) than PR alone. These findings suggest that patient phenotyping, particularly the presence of inspiratory muscle weakness, may be critical in determining the additive value of IMT within PR. Overall, the available literature presents a heterogeneous and sometimes conflicting picture: meta-analytic evidence indicates that IMT is clearly efficacious as a stand-alone intervention, whereas its incremental benefit when layered onto PR appears conditional and context dependet.

The benefits of including IMT in a standard PR training program are still unclear and debatable. This systematic review addresses two core questions: (1) In adults with moderate-to-severe COPD, does adding IMT to PR improve PImax, dyspnea, HRQoL, 6MWT performance, and pulmonary function compared with PR alone? (2) Are the effects of IMT as an adjunct to PR consistent across these outcome domains, or do patient phenotypes and protocol parameters explain heterogeneity?

## Methods

This systematic review followed recommendations proposed by the Cochrane Collaboration ( Higgins and Green, 2011) and the PRISMA Statement ( Moher et al., 2010). The review was registered in PROSPERO (ID: CRD420251251860), and all methods adhered strictly to Cochrane Collaboration standards and PRISMA 2020 guidelines to ensure methodological rigor and reproducibility. The research question used the PICOS strategy (P: subjects diagnosed with chronic obstructive pulmonary diseases; I: inspiratory muscle training; C: pulmonary rehabilitation program; O: inspiratory muscle strength, dyspnea, quality of life, exercise capacity, and PFT; S: RCT, CT, and cohort studies). The review addressed two core questions. First, in adults with COPD, does adding IMT to PR improve PImax, dyspnea scores, HRQoL, 6MWT or ISWT performance, and PFT outcomes compared with PR alone. Second, across eligible RCTs and CTs, are the effects of IMT as an adjunct to PR consistent across these outcome domains.

### Eligibility criteria

Randomized controlled trials (RCT), non-randomized controlled trials (CT), and cohort studies that investigate the effect of IMT with pulmonary rehabilitation in comparison with pulmonary rehabilitation alone were included in this systematic review. Subjects’ criteria include COPD patients diagnosed by spirometry and the stage moderate or above as per GOLD criteria in most participants ( GOLD, 2020). The following outcomes were considered: inspiratory muscle strength, dyspnea, quality of life, exercise capacity, and PFT. Studies with insufficient or incomplete data were excluded.

### Search strategy

Two independent reviewers (FG, MA) searched the following electronic databases: PubMed, ScienceDirect, Cochrane Library, and Web of Science, from their inception to January 2023. The search window was pre-specified at protocol registration and has not been extended for this revision; the implication of this choice are addressed explicitly in the limitation section, together with a narrative comparison to evidence syntheses published subsequently ( Ammous et al., 2023; Xie et al., 2025). The title and abstracts were reviewed by the two reviewers. Further searches were done for the cited references in the article reference list. Any disagreement was resolved by consensus and discussed with the third investigator (SN). Search terms combined subject headings (MeSH/Emtree) and keywords: (“inspiratory muscle training” OR “respiratory muscle training” OR “IMT”) AND (“chronic obstructive pulmonary disease” OR “COPD” OR “chronic obstructive airway disease”) AND (“pulmonary rehabilitation” OR “respiratory rehabilitation” OR “exercise training”). The full electronic search strategies for all databases are provided in the extended data ( *Search strategy*). No restriction was placed on the publication year. Only full-text RCT, CT, and cohort studies in the English language conducted on human subjects and published in peer-reviewed journals were included in this systematic review. Review papers, grey literature, conference proceedings, case studies, and studies using animal subjects or non-COPD participants were excluded.

### Interventions

In line with the PICOS framework, the ‘Intervention’ was IMT delivered via pressure-threshold, flow-resistive, or flow-volumetric devices layered on a supervised pulmonary rehabilitation program. The ‘Comparator’ was pulmonary rehabilitation alone (or, in multi-arm trials, active non-IMT comparators such as cycle ergometer training or expiratory positive-pressure breathing). Detailed protocol-level parameters (device type, % PImax, session and program duration, frequency, and progression rules) are reported at the study level in the Results (see *Table 2* and the ‘Intervention characteristics’ subsection below).

### Data collection process

Data collection were performed by the primary investigator following the standards format. Data included: (1) general characteristics: author’s first name, year of publication, study type; (2) sample: case numbers, intervention/control group, male/female, mean age; (3) program duration: session/week, duration; (4) intervention; (5) PR method; (6) outcome measures: primary (inspiratory muscle strength, “maximal inspiratory pressure PImax”) and secondary (dyspnea “Borg scale,” quality of life, exercise capacity “6MWT, ISWT” and PFT “FEV1/FVC, FEV1, FVC”); (7) Results; (8) Conclusion.

### Risk of bias assessment

The Cochrane collaboration tool to assess the risk of bias for randomization control studies (Rob 2.0) was used for risk bias assessment. Two independent reviewers performed the assessment. The tool has five domains measuring: (1) bias arising from the randomization process, (2) bias due to deviation from intended interventions, (3) bias due to missing outcome data, (4) bias in the measurement of the outcome, and (5) bias in the selection of the reported results. Answers leads to judgments of “low risk of bias” “some concerns,” or “high risk of bias.”

## Results

### Selection of studies

**Figure 1.**
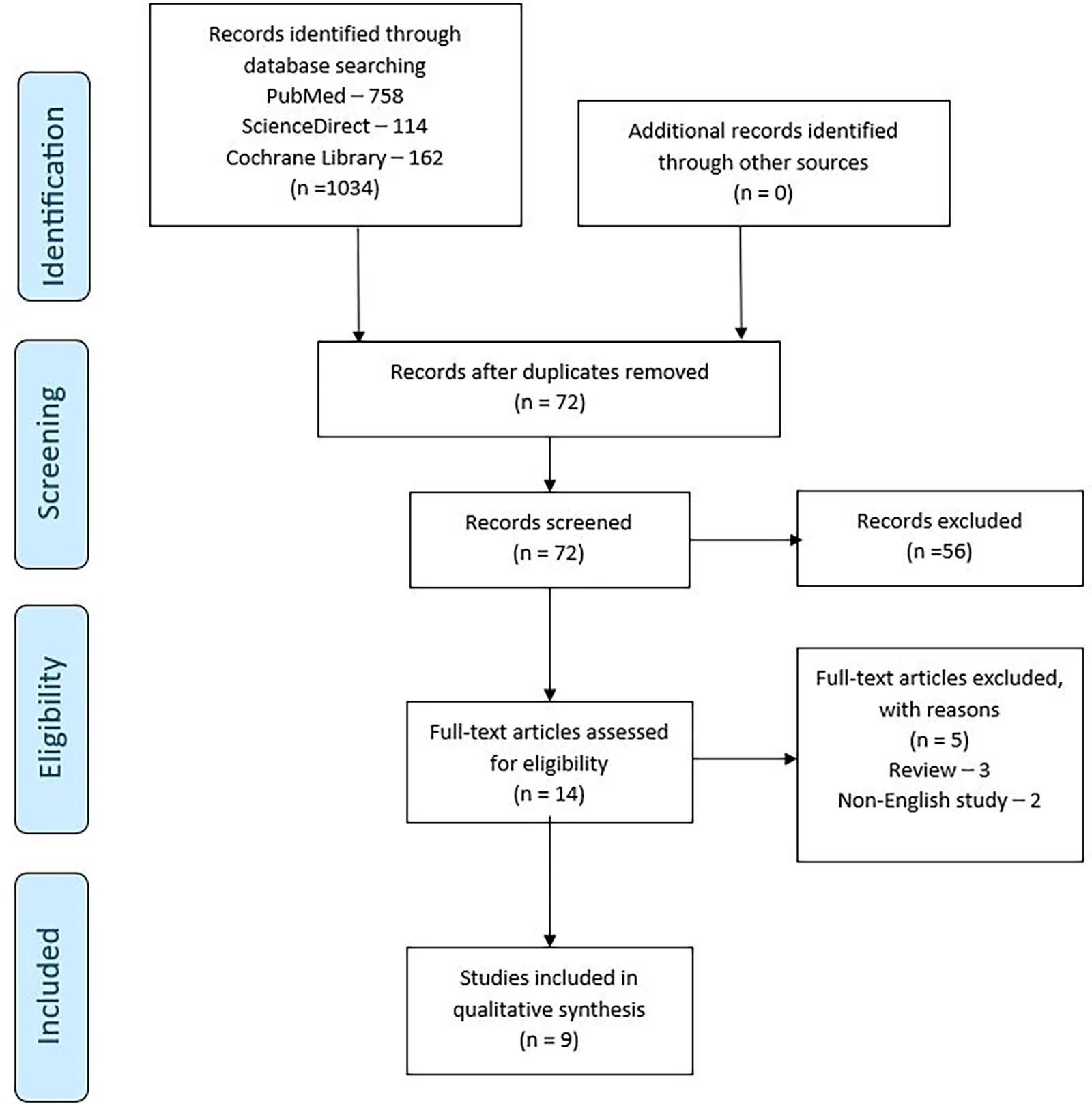
PRISMA flow diagram.

The initial search identified 1,034 records from PubMed, ScienceDirect, Cochrane Library, and Web of Science. After removal of duplicates (n = 962), 72 records were screened at the title/abstract level. Fifty-eight records were excluded at this stage with the following reasons: non-COPD population (n = 19), no IMT intervention (n = 14), wrong study design (n = 12), no pulmonary rehabilitation comparator (n = 8), non-human or in vitro studies (n = 3), and remaining duplicate publications (n = 2). Fourteen full-text reports were sought, retrieved, and assessed for eligibility, of which five were excluded, three review articles and two non-English full texts. Only nine RCT’s studies ( Abedi et al., 2019; Bavarsad et al., 2015; Beaumont et al., 2015; Chuang et al., 2017; Leelarungrayub et al., 2017; Petrovic et al., 2012; Tounsi et al., 2021; Tout et al., 2013; Wang et al., 2017) met the eligibility criteria and were included in this systematic review. Non-randomized studies were also eligible, but none met the final inclusion criteria. The studies selected and the flow chart are shown in Figure 1.

### Study characteristics

The characteristics of the included studies are available as *Table 1* (extended data), with a structured PICOS summary of each trial provided in *Table 2* (extended data). All of the research that was selected was published between 2012 and 2021. These nine studies included a total of 295 individuals. The pressure threshold loading IMT device was the most prevalent type (n = 5). The investigations also used volume-based devices (n = 1) and flow resistive loading devices (n = 2), and in one study, the type of device was not reported (n = 1). The majority of the studies met the PR program’s recommended minimum duration of eight weeks (n = 7). In most studies, either the participants’ genders were not given (n = 3), or the number of male participants was substantially greater (n = 3). In two studies, the participants’ mean ages were in the 50s; for the other studies, the mean age was above 60.

### Study intervention details

**IMT protocols**: Five of nine trials used pressure-threshold loading devices (Threshold IMT, Respifit S, or generic pressure-threshold loaders) ( Beaumont et al., 2015; Chuang et al., 2017; Petrovic et al., 2012; Tout et al., 2013; Wang et al., 2017). Two used flow-resistive devices (PowerBreathe in Tounsi et al., 2021, and standard and prototype resistive devices in Leelarungrayub et al., 2017). One used a flow-volumetric inspiratory exerciser (Respivol in Bavarsad et al., 2015), and one did not specify device type ( Abedi et al., 2019). Training intensity was most commonly prescribed as 30% of baseline PImax with progressive increase ( Wang et al., 2017; Tout et al., 2013 progressing to 60% PImax). Tounsi et al. (2021) progressed from 50% to 80% PImax every 2 weeks; Petrovic et al. (2012) trained at ≥80% PImax for strength and 60-70% PImax for endurance; Chuang et al. (2017) started at 15 cmH _2_O absolute load and progressed every 2 weeks; and Leelarungrayub et al. (2017) progressed resistive diameter from 6 mm to 4 mm to 2 mm. Session duration ranged from 15 min ( Bavarsad et al., 2015) to 30 min ( Beaumont et al., 2015; Chuang et al., 2017). Frequency ranged from 5 days/week ( Chuang et al., 2017) to 7 days/week ( Petrovic et al., 2012; Tounsi et al., 2021; Leelarungrayub et al., 2017), and total program duration from 3 weeks ( Beaumont et al., 2015) to 8 weeks in the remaining trials. All active IMT arms included at least once-daily supervised or home-based training with periodic supervision for load adjustment.

**Pulmonary rehabilitation programs**: The PR backbone varied in reported granularity. Aerobic training was most frequently prescribed as cycle-ergometer or treadmill endurance, 20-30 min per session, 3-5 sessions/week, at 60-80% of the speed or work rate derived from the 6-minute walk test or incremental symptom-limited exercise test ( Tounsi et al., 2021: 30 min treadmill at 60-80% 6MWT average speed, 3 sessions/week; Wang et al., 2017: cycle ergometer 20-30 min, 3-5 sessions/week; Petrovic et al., 2012: constant-load cycle testing at 75% peak work rate). Resistance training was reported in Beaumont et al. (2015), Tout et al. (2013), and Wang et al. (2017), typically as upper- and lower-limb strengthening at 60-80% of 1-RM (or equivalent self-rated exertion), 2-3 sets of 8-12 repetitions, 2-3 sessions/week, with progressive load adjustment. Program duration ranged from 3 weeks ( Beaumont et al., 2015, inpatient PR) to 12 weeks (elective outpatient program in Abedi et al., 2019 and Tout et al., 2013), with the majority (seven of nine trials) meeting the internationally recommended minimum PR duration of 8 weeks. Educational components (inhaler technique, energy conservation, airway clearance, smoking cessation, and self-management) were reported in six of nine trials.

### Methodological quality

**Figure 2.**
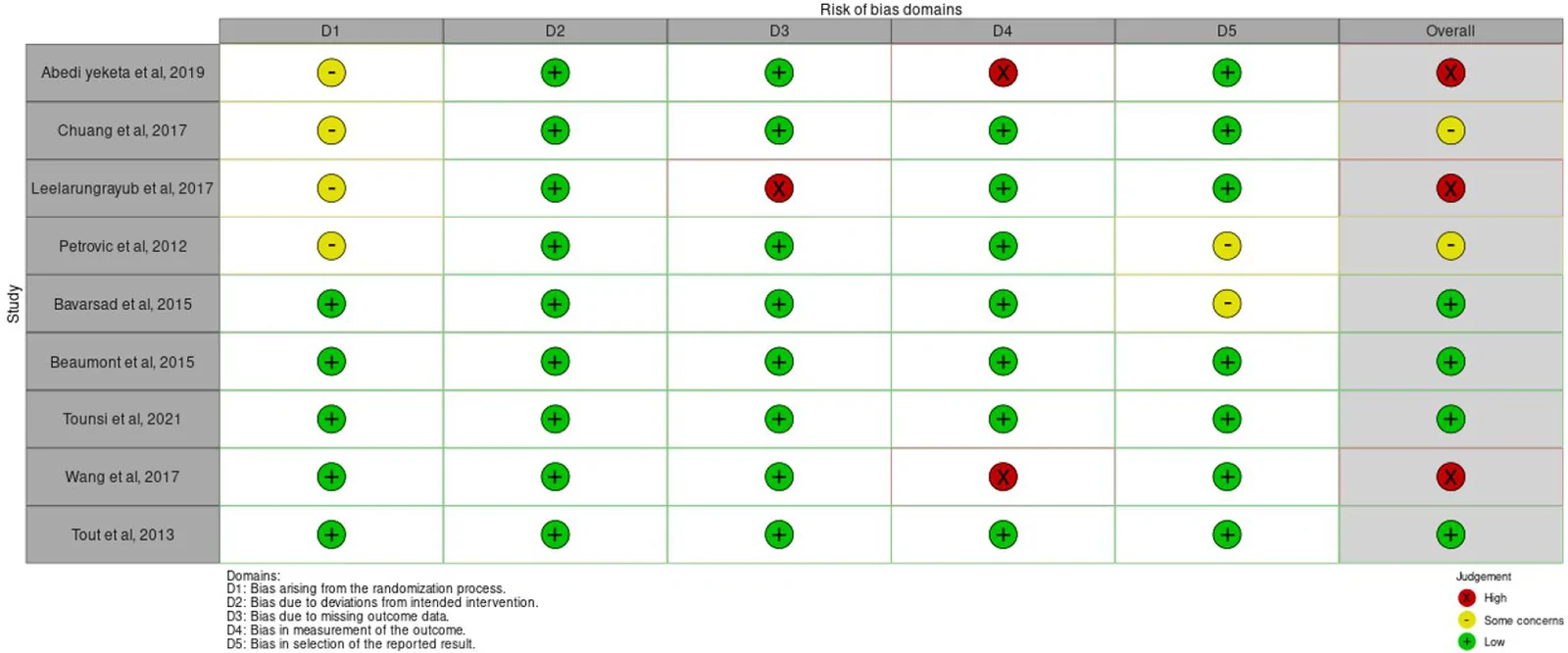
Risk of bias.

The Rob 2.0 scale was used to evaluate the risk of bias in the chosen studies. All of the studies included were described as randomized, and the baseline between the two randomization arms appears to be balanced. Three studies ( Abedi et al., 2019; Leelarungrayub et al., 2017; Wang et al., 2017) were rated as high risk of bias due to lack of blinded outcome assessment for patient-reported outcomes (dyspnea, quality of life), which may have inflated treatment effects ( Figure 2). Overall, 4 studies had low risk, 2 had some concerns, and 3 had high risk across all RoB 2.0 domains.

## IMT effects on the outcome measures

A detailed summary of outcome effects across included studies is provided in *Table 3* in the extended data.

### Inspiratory muscle strength (PImax)

Inspiratory muscle strength was reported in seven studies: Beaumont et al. (2015), Chuang et al. (2017), Leelarungrayub et al. (2017), Petrovic et al. (2012), Tout et al. (2013), Wang et al. (2017), and Tounsi et al. (2021). Six studies (85.7%) demonstrated statistically significant PImax improvements following IMT. Petrovic et al. (2012) reported an increase of 14.0 cmH _2_O (77.5 ± 4.7 to 91.5 ± 5.2 cmH _2_O; p < 0.001), while Tounsi et al. (2021) documented an increase of 22.9 ± 5.8 cmH _2_O (61.9 ± 21.8 to 84.8 ± 20.9 cmH _2_O; p < 0.001). Chuang et al. (2017) observed an improvement of 17.6 ± 0.18 cmH _2_O (p < 0.001) compared to a small change of 2.21 ± 0.4 cmH _2_O in controls after 8 weeks of threshold IMT. Wang et al. (2017) reported a modest but significant increase of 5.20 ± 0.89 cmH _2_O (p < 0.001) in participants receiving combined cycle ergometer training and IMT. Leelarungrayub et al. (2017) demonstrated significant PImax increases in both the standard threshold group (54.0 ± 5.16 to 84.0 ± 7.07 cmH _2_O; p = 0.007) and prototype device group (53.50 ± 5.20 to 83.6 ± 4.40 cmH _2_O; p < 0.001), with no change in the control group. The IMT group in Tout et al. (2013) also reported significant PImax increases (p = 0.008).

Two studies reported no significant PImax changes. Bavarsad et al. (2015) showed no improvement despite gains in exercise capacity and dyspnea, suggesting that mechanisms beyond inspiratory muscle strengthening contribute to clinical outcomes. Beaumont et al. (2015) enrolled patients with preserved baseline PImax (80 ± 7 cmH _2_O, 95% predicted), indicating a ceiling effect, patients with baseline inspiratory muscle weakness derive greater benefit from IMT than those with preserved function.

The magnitude of PImax improvements ranged from 5.2 to 22.9 cmH _2_O, with most exceeding established MCID thresholds. Heterogeneity in responses appears influenced by baseline strength, device characteristics, intervention duration, and training intensity. Studies employing higher-intensity protocols ( Tounsi et al., 2021: 50% to 80% PImax; Petrovic et al., 2012: ≥80%) achieved larger absolute gains.

### Expiratory muscle strength (PEmax)

Expiratory muscle strength was assessed in only three studies. Leelarungrayub et al. (2017) reported that PEmax improved significantly in both the standard and prototype device groups, while the control group showed no significant change. Wang et al. (2017) presented ΔPEmax values of −5.29 ± 1.97 cmH _2_O in the control group, 5.42 ± 1.92 cmH _2_O in the CET group, and 2.37 ± 1.88 cmH _2_O in the combined CET+IMT group (p = 0.001), indicating both intervention groups were superior to control. However, Tout et al. (2013) found no significant change in PEmax in any group.

These findings suggest that standard IMT protocols predominantly target inspiratory musculature and do not substantially enhance expiratory muscle function.

### Dyspnea

Dyspnea was assessed in seven studies using validated instruments including the mMRC scale, Borg category-ratio scale, BDI/TDI, and MDP questionnaires. six studies reported within-group dyspnea improvement in at least one active group; however, between-group superiority for IMT was not consistently demonstrated. Bavarsad et al. (2015) showed Borg scale improvement from 3.76 ± 2.49 to 1.13 ± 1.39 (p < 0.0001), a reduction of approximately 2.63 points, exceeding the established MCID of 1.0 point. Petrovic et al. (2012) reported Borg CR10 reduction from 5.0 ± 1.0 to 4.0 ± 1.1 (p < 0.01), and during constant-load testing from 7.0 ± 0.7 to 5.0 ± 0.9 (p < 0.001). Chuang et al. (2017) demonstrated BDI/TDI improvement from 4.48 ± 2.12 to 9.0 ± 2.27 (p < 0.001), indicating substantial clinical change.

Tout et al. (2013) showed that all four groups (IMT, PEP, IMT+PEP, and control) improved significantly on the Sadoul scale, with no between-group differentiation. Wang et al. (2017) reported greater improvements in mMRC and CAT scores in both intervention groups compared to control.

Leelarungrayub et al. (2017) revealed a nuanced pattern: while peak dyspnea during maximal exercise increased slightly, resting dyspnea and dyspnea at standardized workloads decreased significantly (p < 0.006). This suggests improved ventilatory efficiency, where patients may perceive greater respiratory sensation at maximum effort but experience reduced dyspnea during submaximal activities. Beaumont et al. (2015) reported modest improvement, possibly due to the short intervention duration (3 weeks) and preserved baseline inspiratory function.

The mechanisms underlying dyspnea reduction appear multifactorial, including improvements in inspiratory muscle strength and endurance, reductions in dynamic hyperinflation, and enhanced self-efficacy. The consistency across diverse populations, protocols, and instruments suggests dyspnea reduction represents a robust IMT outcome.

### Exercise capacity (6-minute walk test)

Seven studies evaluated exercise capacity using 6MWT. Four studies demonstrated statistically significant within-group improvements. Bavarsad et al. (2015) increased 6MWT distance by 45.46 meters (445.6 ± 89.05 to 491.06 ± 93.8 meters; p < 0.0001), exceeding the established MCID of 25-30 meters. Chuang et al. (2017) reported improvement of 47.8 ± 1.46 meters (p < 0.001).

Between-group comparisons revealed heterogeneous patterns. Beaumont et al. (2015) found no significant between-group differences in 6MWT improvements, with both IMT and control groups showing modest gains (p = 0.7). Wang et al. (2017) reported significant between-group differences Δ6MWD was -1.64 ± 4.64 m in controls, 32.55 ± 4.59 m in the CET group, and 21.68 ± 4.51 m in the combined group; between-group comparison was significant (p < 0.001). Tounsi et al. (2021) provided evidence for time-dependent effects: at 4-week assessment, no significant between-group differences emerged (p = 0.92), but by 8 weeks, the IMT+endurance training group demonstrated substantially greater improvement (42.6 ± 9.8 versus 29.8 ± 7.4 meters), suggesting IMT benefits for exercise capacity may require adequate duration to manifest.

Overall, four studies demonstrated within-group improvements meeting the MCID threshold (≥25 meters). However, the inconsistent between-group superiority of PR+IMT over PR alone suggests that while IMT produces meaningful absolute improvements, these often occur similarly in standard PR, indicating that additional IMT benefit may be modest or time-dependent.

### Quality of life

Health-related quality of life (HRQoL) was assessed in five studies using SGRQ, SF-36, CCQ, ABC, and BBS instruments. All five studies (100%) reported statistically significant HRQoL improvements following IMT. Abedi et al. (2019) showed SGRQ total score improvement after 8 weeks in all groups, with the greatest change in the combined IMT+aerobic group (Δ5.5 ± 3.54; p < 0.001). Chuang et al. (2017) reported substantial improvement in SF-36 physical component score (24.58 ± 20.54; p < 0.001) and mental component score (26.14 ± 22.24; p < 0.001), both exceeding established MCID thresholds (5–10 points). Leelarungrayub et al. (2017) demonstrated significant improvements across all CCQ domains in both device groups (p < 0.05). Tout et al. (2013) found significant SGRQ improvements in all groups (IMT, PEP, IMT+PEP, and control), with no clear superiority of any active modality. Wang et al. (2017) reported greater SGRQ improvements in CET (−3.51 ± 0.54) and combined groups (−3.32 ± 0.54) compared to control (0.95 ± 0.56), with significant between-group differences (p < 0.001).

The universal HRQoL improvement across all studies contrasts with the more variable exercise capacity findings, suggesting that patient-perceived benefits may surpass objective functional gains measured by performance-based tests.

### Pulmonary function tests (FEV _1_ and FVC)

Spirometric parameters were assessed in four studies. Bavarsad et al. (2015) and Wang et al. (2017) reported no changes in any pulmonary function measures (FEV _1_, FVC, FEV _1_/FVC, FEF25–75) in either group. Similarly, Tout et al. (2013) observed statistically significant improvements only in the IMT-only group, where FEV _1_ increased from 0.93 ± 0.39 to 1.44 ± 0.57 L (p = 0.03) and PEFR from 0.57 ± 0.14 to 0.76 ± 0.16 L (p = 0.01); all other groups and PFT measures were non-significant. Leelarungrayub et al. (2017) reported increases in FVC and FEV _1_/FVC ratio in both standard and prototype device groups, suggesting potential improvements in ventilatory mechanics without absolute FEV _1_ changes, possibly reflecting reduced dynamic hyperinflation.

The consistent absence of substantial spirometric improvement has important mechanistic implications. Structural airway resistance from fibrosis and alveolar destruction cannot be reversed by IMT, which targets respiratory muscle performance rather than fixed airway obstruction. This dissociation confirms that IMT primarily operates at the neuromuscular level, serving as a symptomatic rather than disease-modifying intervention.

### Dynamic hyperinflation and exercise endurance

Petrovic et al. (2012) provided mechanistic insight by evaluating dynamic hyperinflation parameters. During the constant-load test at 75% peak work rate, exercise time increased from 597.1 ± 80.8 to 733.6 ± 74.3 seconds (22.9% increase; p < 0.001). Inspiratory muscle endurance (tlim) increased from 348 ± 54 to 467 ± 58 seconds (34% increase; p < 0.001). Inspiratory fraction (IF) increased significantly in both incremental (0.41 ± 0.05 to 0.45 ± 0.05; p < 0.001) and constant-load tests (0.43 ± 0.03 to 0.44 ± 0.03; p < 0.001), indicating meaningful reduction in dynamic hyperinflation.

Wang et al. (2017) reported improvement in inspiratory capacity (IC) in both intervention groups relative to control: 0.06 ± 0.02 L in the CET group, and 0.10 ± 0.02 L in the combined group (p < 0.001). These findings demonstrate that IMT benefits extend beyond static assessments to functional exercise performance and mechanistic parameters of respiratory limitation.

### Summary of outcome patterns

A clear pattern emerges across nine studies and multiple outcome domains. PImax improved significantly in 85.7% of studies, with non-responders generally demonstrating ceiling effects from preserved baseline function. Dyspnea reduction represented one of the most responsive patient-centered outcomes, with all studies showing within-group improvement, although between-group superiority was inconsistent. Exercise capacity improvements were less consistently significant in between-group comparisons, though 71.4% of studies reported within-group improvements that met MCID thresholds. HRQoL demonstrated uniform improvement (100%), suggesting robust patient-perceived benefits despite variable objective measures. Spirometric indices remained largely unchanged, with only isolated improvement in selected parameters, confirming that IMT does not modify underlying fixed airflow obstruction.

The specific protocol-level sources of this variability (baseline PImax, training intensity (% PImax), session and programme duration, device type, and comparator intensity) are detailed in Table 2 and are referenced directly in the Discussion’s treatment of heterogeneity.

Heterogeneity appears attributable to: (1) baseline inspiratory muscle strength, with weaker patients showing greater improvement potential; (2) device characteristics; (3) patient selection criteria, particularly confirmed inspiratory muscle weakness versus unselected cohorts; (4) intervention duration and training intensity; (5) integration with standard pulmonary rehabilitation; and (6) methodological rigor. Understanding these sources of heterogeneity is essential for clinicians designing future IMT protocols and for interpreting findings within individual patient contexts.

## Discussion

The principal finding of this systematic review is that adding IMT to pulmonary rehabilitation for moderate-to-severe COPD consistently improves inspiratory muscle strength and health-related quality of life, while effects on dyspnea and exercise capacity are clinically meaningful but more variable in between-group comparisons. These findings establish IMT as a patient-centered adjunct that primarily addresses symptomatic burden and quality of life, even in the absence of consistent improvements in walk distance or spirometry beyond standard rehabilitation.

Patients with moderate-to-severe COPD commonly exhibit inspiratory muscle weakness that is both neuromechanical (shortening of the diaphragm at high operating lung volumes secondary to hyperinflation) and myopathic (reduced type I fibre density, sarcomeric remodelling, activation of the ubiquitin-proteasome proteolytic pathway) ( Levine et al., 1997; Ottenheijm et al., 2006). The resulting reduction in PImax elevates the ratio of required-to-available inspiratory pressure during exertion, which is a central afferent signal for the perception of dyspnea and a key driver of exercise cessation ( Laveneziana et al., 2015). Hyperinflation further compounds this disadvantage by shifting the diaphragm onto a suboptimal portion of its length-tension curve and by fixing end-expiratory lung volume above functional residual capacity during exercise, generating dynamic hyperinflation and early tidal-volume constraint ( Rossi et al., 2015; O’Donnell et al., 2009). IMT counteracts these mechanisms by producing fibre-type remodelling, increasing diaphragmatic cross-sectional area and PImax, and, as directly demonstrated by Petrovic et al. (2012) in our review, reducing dynamic hyperinflation as indexed by an increase in inspiratory fraction during incremental and constant-load exercise. This mechanistic chain provides a coherent explanation for our observation that PImax and dyspnea are the most consistently improved outcomes after IMT, while spirometric indices (which reflect fixed airflow obstruction rather than inspiratory muscle function) are essentially unchanged.

Our systematic review aligns with the conclusions of the contemporaneous Cochrane review ( Ammous et al., 2023). Using different search strategies, both reviews converge on the same pattern: adding IMT to PR significantly improves PImax (ranging from 5.2 to ~30 cmH _2_O in our review) but does not consistently improve dyspnea or exercise capacity significantly beyond PR alone. This agreement strengthens the evidence that while routine addition of IMT to all PR programs may not be necessary, it provides distinct benefits for specific outcomes. Our review extends these findings by demonstrating that 100% of the six included studies measuring quality of life reported statistically significant improvements, indicating a strong patient-centered signal that appears more consistent than functional exercise outcomes.

Prior systematic reviews, such as Beaumont et al. (2018), which found no added effect of IMT on dyspnea during PR, noted conflicting evidence for combined interventions. In this review, the inclusion of recent RCTs with diverse protocols reveals a detailed picture: IMT confers the greatest benefit when targeted to patients with specific deficits or when using sufficiently intensive protocols ( Tounsi et al., 2021, using 50–80% intensity). Importantly, while exercise capacity improved within groups in the majority of studies (57.1%), the inconsistent superiority of PR+IMT over PR alone suggests that the additional functional gain from IMT may be limited when a comprehensive rehabilitation program is already in place.

The physiological rationale for these improvements centers on neuromuscular adaptations. IMT enhances inspiratory muscle strength (PImax) and endurance, as evidenced by significant gains in all seven studies reporting this outcome. Mechanistically, studies documenting improvements in inspiratory capacity ( Wang et al., 2017) and reductions in dynamic hyperinflation ( Petrovic et al., 2012) support the role of IMT in enhancing operational lung volumes, potentially reducing the sense of breathlessness for a given workload. These adaptations likely underlie the uniform benefits seen for quality of life, even in the absence of changes in spirometric indices (FEV _1_ or FVC), which remained unchanged in nearly all studies. This dissociation confirms that IMT operates as a symptomatic intervention targeting respiratory muscle performance rather than modifying fixed airway obstruction.

Heterogeneity in effect estimates across the nine included trials is not random but systematically linked to the protocol-level parameters reported in Table 2. First, baseline inspiratory muscle strength varied widely, from PImax ≈61 cmH _2_O in Tounsi et al. (2021) to preserved function (mean 80 cmH _2_O, 95% predicted) in Beaumont et al. (2015), predicting greater response in the former. Second, training intensity ranged from 15 cmH _2_O absolute load ( Chuang et al., 2017) to ≥80% PImax ( Petrovic et al., 2012; Tounsi et al., 2021 at 8 weeks), with larger effect sizes observed at higher intensities. Third, program duration varied between 3 and 12 weeks, with the shortest program ( Beaumont 2015) showing the smallest between-group signal. Fourth, device types spanned pressure-threshold, flow-resistive, and flow-volumetric loaders, each with different load-velocity characteristics. Fifth, comparator intensity varied: trials in which the comparator PR program already included vigorous whole-body endurance training ( Wang et al., 2017; Tounsi et al., 2021 endurance-training arm) produced smaller relative IMT increments than trials with less intensive PR comparators. Recognizing heterogeneity as structured rather than random reframes the clinical question from “does IMT work?” to “for which patients, at which intensity, for how long, compared with which PR backbone?” — a question the present review addresses narratively and that a future individual-patient-data meta-analysis will be required to answer definitively.

The overall strength of this evidence is moderate. While inspiratory muscle strength and quality of life showed consistent positive signals, precision was limited by generally small sample sizes and short intervention durations (typically 8 weeks or less). Risk of bias was significant in approximately one-third of studies, primarily due to lack of assessor blinding for patient-reported outcomes. However, the coherence of findings across diverse settings, especially the universal improvement in quality of life, supports the reliability of the main conclusions.

Clinically, these findings argue for the selective inclusion of IMT in pulmonary rehabilitation. It is most strongly indicated for patients with confirmed inspiratory muscle weakness, or those who remain highly symptomatic with poor quality of life despite standard therapy. Programmatic implications include the need for baseline PImax assessment to identify responders and the use of progressive, high-intensity protocols to maximize strength gains. From a clinical implementation perspective, the data support IMT as a high-value adjunct for targeted populations rather than a mandatory component for all COPD patients.

Why IMT layered on PR can exceed PR alone is best understood through a dose-response and phenotype-matching lens. Standard PR targets locomotor muscles and cardiorespiratory fitness; the inspiratory pump is loaded only to the extent demanded by whole-body exercise, which is typically sub-threshold for strength adaptation in weakened inspiratory muscles. Dedicated IMT provides a specific, progressive, and supra-threshold load on the diaphragm and accessory inspiratory muscles, generating adaptations that general exercise does not reliably elicit. Consistent with this, the trials in our review that delivered the highest training intensity and longest progression ( Petrovic et al., 2012 at ≥80% PImax for 8 weeks; Tounsi et al., 2021 progressing from 50% to 80% PImax over 8 weeks) achieved the largest PImax gains and the most consistent mechanistic endpoints (reduced dynamic hyperinflation and improved breathing pattern). Conversely, trials that enrolled patients without inspiratory muscle weakness ( Beaumont et al., 2015, baseline PImax ≈95% predicted) observed little incremental benefit from IMT, consistent with a ceiling effect. This is precisely the pattern predicted by the current mechanistic model: IMT has its greatest additive value when (a) inspiratory muscle weakness is present at baseline (PImax <60 cmH _2_O or <50% predicted), (b) the training load is progressed into a hypertrophic range (≥60% PImax), and (c) the program is sustained for at least 8 weeks. This phenotype-protocol framework explains, without requiring appeal to publication bias or measurement artefact, why the aggregate between-group signal for IMT + PR is robust for PImax and dyspnea but attenuated for 6MWT performance.

### Limitations

This systematic review has several limitations. First, the small sample sizes of individual studies limited statistical power to consistently detect between-group differences. Second, substantial methodological heterogeneity prevented formal meta-analysis: protocols varied across device type, training intensity, and duration. Third, the lack of long-term follow-up restricts conclusions about the durability of IMT benefits. Fourth, measurement inconsistency, with diverse tools used for dyspnea and quality of life, complicated cross-study comparison. Fifth, the temporal scope of the review represents an important constraint: the literature search closed in January 2023 and was not re-run for this revision in order to preserve the pre-specified screening, and risk-of-bias. As a result, we cannot directly account for IMT trials published between February 2023 and 2026, including those evaluating emerging devices such as electronic tapered-flow resistive loaders and telemonitored resistive loaders. Reassuringly, two evidence syntheses published after our closing date ( Ammous et al., 2023; Xie et al., 2025) report directionally concordant findings, including significant PImax gains, inconsistent between-group superiority for dyspnea and exercise capacity, and modest effects on spirometry, suggesting that our substantive conclusions are unlikely to be overturned by more recent evidence. Finally, the exclusion of non-English studies and potential publication bias inherent in small trials may influence the generalizability of findings. Despite these limitations, the consistent signal for patient-centered benefit supports the clinical utility of IMT in appropriate contexts.

## Conclusions

This systematic review provides moderate-quality evidence that inspiratory muscle training, when incorporated into pulmonary rehabilitation programs, yields consistent improvements in patient-centered outcomes for individuals with moderate-to-severe COPD. Inspiratory muscle strength and dyspnea demonstrate significant improvements in six of seven (85.7%) and all seven (100%) reporting studies respectively, while health-related quality of life shows universal enhancement across all assessing studies. Exercise capacity benefits, though more variable in between-group comparisons, frequently meet clinically meaningful thresholds within intervention groups. Spirometric indices remain largely unaffected, confirming IMT’s targeted mechanism on respiratory muscle function rather than fixed airflow obstruction. The evidence indicates that IMT provides greatest benefit when targeted to patients with confirmed inspiratory muscle weakness, escalated to progressive intensity (starting 30–50% PImax, advancing to 60–80%), and sustained for adequate duration (≥8 weeks). While methodological heterogeneity limits definitive protocol optimization, the cumulative evidence supports selective IMT integration into pulmonary rehabilitation for appropriately phenotyped patients. Future research priorities include adequately powered, multicenter trials with standardized protocols, extended follow-up to assess durability, comparative evaluations of different device types and intensity regimens, mechanistic studies linking dynamic hyperinflation reduction to symptom benefits, and cost-effectiveness analyses to inform implementation strategies. Clinicians should consider baseline inspiratory muscle assessment, individualized IMT prescription, and monitoring of adherence when incorporating IMT into comprehensive COPD rehabilitation programs.

## Ethics and consent

Ethical approval and consent were not required.

## Data Availability

All data underlying the results are included in this article; no new datasets were generated. Extended data (Extended data: Search strategy and study selection, and the PRISMA 2020 checklist) are available on Figshare: https://doi.org/10.6084/m9.figshare.30957968 ( Algharbi et al., 2025). Data are available under the terms of the Creative Commons Attribution 4.0 International license.data waiver ( CC BY 4.0 Public domain dedication). The PRISMA 2020 checklist for this systematic review is included in the Extended data on Figshare (see Data availability statement) https://doi.org/10.6084/m9.figshare.30957968 ( Algharbi et al., 2025). Data are available under the terms of the Creative Commons Attribution 4.0 International license data waiver ( CC BY 4.0 Public domain dedication).
