# Supplementary material for: Inspiratory Muscle Training plus Pulmonary Rehabilitation versus Rehabilitation alone in COPD A Systematic Review of Randomized Controlled Trials
Source: F1000Res. 2026 May 5;15:19. Originally published 2026 Jan 7. [Version 3] doi: 10.12688/f1000research.175598.3 (PMC13555003; doi:10.12688/f1000research.175598.3)
Supplement: Peer review history

# Reviewer report: Reviewer response for version 3

**DOI:** 10.5256/f1000research.200338.r498181

**Published:** 2026-07-24

**Authors:** Ivanizia S. Silva

**Affiliations:** 1 Hospital Municipal de Natal, Natal, Rio Grande do Norte, Brazil; 2 Department of Physical Therapy, Federal University of Rio Grande do Norte, Natal, Brazil

Thank you for the opportunity to review the manuscript entitled “Inspiratory Muscle Training plus Pulmonary Rehabilitation versus Rehabilitation alone in COPD A Systematic Review of Randomized Controlled Trials”.

 The manuscript aims to analyze whether IMT plus PR provides benefits beyond PR alone in adults with moderate to severe COPD, evaluate the impact of IMT use on PImax, dyspnea, HRQoL, 6MWT, and pulmonary function tests (PFTs).

 I have provided several comments below that I believe will help improve the scientific rigor and overall quality of the manuscript.

 1. Introduction

 a. The authors mentioned two studies ( Beaumont et al., 2018; Ammous et al., 2023) that reported that adding IMT to PR increased PImax, but did not significantly enhance dyspnea, quality of life, or exercise capacity when compared with PR alone. However, the significance of this review is unclear. What does it contribute to the current evidence?

 2. Methods

 a. The registration in PROSPERO was performed in December 2025. However, the search in scientific databases was conducted in January 2023. Moreover, there is an important temporal lapse. The author reassures that only two evidence syntheses (Ammous et al., 2023; Xie et al., 2025) are published after their closing date. Thus, I strongly suggest the inclusion of these two trials in the systematic review.

 b. It is possible that any unpublished studies identified in a given review may be an unrepresentative subset of all the unpublished studies in existence. However, the bias this introduces is of less concern than the bias introduced by excluding all unpublished studies. Likewise, while searching for, and analyzing, studies in any language can be extremely resource-intensive, review authors should consider carefully the implications for bias. Therefore, I suggest that the authors justify why they excluded grey literature and non-English language studies.

 c. The Review authors should specific metric used to characterize each participant’s results (e.g. post-intervention or change from baseline). 

 3. Results

 a. I am not sure if POWERbreathe is a flow-resistive device.

 b. Abedi et al., 2019 did not specify device type used for IMT. Review authors often find that they are unable to obtain all the information they seek from available reports. In such circumstances, authors are strongly encouraged to contact the original investigators. 

 c. Why wasn't the meta-analysis performed? If there was substantial heterogeneity among the studies, making it impossible to combine them into a meta-analysis, this should be better described. What I2 value was considered to represent substantial heterogeneity?

 d. What is the established MCID threshold for Pimax?

 e. I found the wording of the results particularly difficult to understand. The pre- and post-intervention comparison does not reflect the effect of the intervention. The authors should report the comparison between the intervention and control groups.

 4. Conclusion

 a. Based on the above considerations, it is likely that the conclusion needs to be adjusted.

Are the rationale for, and objectives of, the Systematic Review clearly stated?

Partly

Is the statistical analysis and its interpretation appropriate?

No

If this is a Living Systematic Review, is the ‘living’ method appropriate and is the search schedule clearly defined and justified? (‘Living Systematic Review’ or a variation of this term should be included in the title.)

Not applicable

Are sufficient details of the methods and analysis provided to allow replication by others?

Partly

Are the conclusions drawn adequately supported by the results presented in the review?

Partly

Reviewer Expertise:

I am a physiotherapist with experience in systematic reviews and studies on IMT.

I confirm that I have read this submission and believe that I have an appropriate level of expertise to confirm that it is of an acceptable scientific standard, however I have significant reservations, as outlined above.

# Reviewer report: Reviewer response for version 3

**DOI:** 10.5256/f1000research.200338.r481839

**Published:** 2026-05-27

**Authors:** Patsaki Irini

**Affiliations:** 1 University of West Attica, Egaleo, Greece

The authors have addressed all comments and issues raised. They went through an extended revision of their manuscript and enriched their manuscript.

Are the rationale for, and objectives of, the Systematic Review clearly stated?

Yes

Is the statistical analysis and its interpretation appropriate?

Not applicable

If this is a Living Systematic Review, is the ‘living’ method appropriate and is the search schedule clearly defined and justified? (‘Living Systematic Review’ or a variation of this term should be included in the title.)

No

Are sufficient details of the methods and analysis provided to allow replication by others?

Yes

Are the conclusions drawn adequately supported by the results presented in the review?

No

Reviewer Expertise:

An extended research interest in IMT.

I confirm that I have read this submission and believe that I have an appropriate level of expertise to confirm that it is of an acceptable scientific standard.

# Reviewer report: Reviewer response for version 2

**DOI:** 10.5256/f1000research.197361.r470806

**Published:** 2026-04-21

**Authors:** Patsaki Irini

**Affiliations:** 1 University of West Attica, Egaleo, Greece

Algharbi and co- authors have investigated the effectiveness of inspiratory muscle training along with pulmonary rehabilitation in COPD population. As IMT is a well established intervention that could alleviate symptoms in this population and positively effect exercise capacity and quality of life, the authors have tried to underline that should be included in pulmonary rehabilitation as this exceeds the benefits of a stand alone rehabilitation program. COPD patients could manifest significant inspiratory muscle weakness that restricts their functionality, as it limits their exercise capacity. Thus IMT is proposed as the most suitable intervention, and this supports the hypothesis of the authors.

 The manuscript is well written, and its methodology is well presented. The authors have described in detail their findings in all outcomes investigated.

 Major concerns.

 1. its main limitation is that included studies up to January 2023. And this is my major concern as since then, we have more published studies on this topic and the use of new devices and techniques regarding IMT.

 2. The authors need to present the reasons for all excluded studies in figure 1 for reasons of transparency.

 3. There is a need for the authors to include a table following the PICOs framework, presenting all information of the included studies with the outcomes included in each study, along with the main findings in relation to p value. This is most important in order to have an overall idea of the included studies, their interventions and their findings.

 4. A full description of the IMT protocols and the pulmonary rehabilitation programs especially in terms of the aerobic and strength training programs. These will allow us to better understand and explain the findings. This will also improve the discussion of this manuscript.

 5. The discussion needs to be enhanced. The authors could add further support of their findings in relation to the interventions applied.

 Minor concerns.

 1. The authors seems that described the characteristics of the interventions in the methodology section in relation to what they found, but this should better be placed at the results. Although there is need to present the interventions investigated under the PICOs framework, but a detailed description exceeds this concept.

 2. The authors have stated the presence of heterogeneity, yet as we don’t have full description of the studies, this limits our understanding of the matter.

 This is a significant study that could lead to further clinical recommendations. The authors have gone through a thorough search in quite few databases and have presented the quality and risk of bias of the included studies. The main concern is the limitation of the year that the search was closed.

Are the rationale for, and objectives of, the Systematic Review clearly stated?

Yes

Is the statistical analysis and its interpretation appropriate?

Not applicable

If this is a Living Systematic Review, is the ‘living’ method appropriate and is the search schedule clearly defined and justified? (‘Living Systematic Review’ or a variation of this term should be included in the title.)

No

Are sufficient details of the methods and analysis provided to allow replication by others?

Yes

Are the conclusions drawn adequately supported by the results presented in the review?

No

Reviewer Expertise:

An extended research interest in IMT.

I confirm that I have read this submission and believe that I have an appropriate level of expertise to confirm that it is of an acceptable scientific standard, however I have significant reservations, as outlined above.

# Response

**Published:** 2026-04-24

**Authors:** Fahad Algharbi

**Affiliations:** College of Applied Medical Sci, Imam Abdulrahman bin Faisal University, Dammam, Saudi Arabia

**We are sincerely grateful to Dr. Patsaki Irini for her careful, constructive, and expert review. Her comments reflect deep clinical and methodological familiarity with respiratory rehabilitation and inspiratory muscle training (IMT) in chronic obstructive pulmonary disease (COPD), and have directly improved the precision, transparency, and clinical utility of our manuscript. We also appreciate her thoughtful and generally positive assessment of our methodology and outcome reporting.**

**Answer 1 (Major Concern 1, Search window ending January 2023)**

 Thank you for foregrounding this important point. We agree that an ideal systematic review would reflect the most recent literature, and we accept this as a genuine limitation. After careful deliberation, a protocol-locked search update was not feasible within the revision timeline, as the statistical analysis, and risk-of-bias assessment were pre-specified against the closed January 2023 evidence base. Re-running the full multi-reviewer screening, data-extraction, and RoB 2.0 workflow would require a new a priori protocol to preserve methodological integrity.

 We note three points supporting the continued validity of our synthesis: (i) The review by Ammous et al. (2023), reached convergent conclusions, significant PImax gains with less consistent dyspnea/6MWT superiority; (ii) Xie et al. (2025), extending through 2024, confirms stable directionality despite mixed exercise capacity signals; (iii) Our phenotype-protocol conclusions (baseline weakness, ≥60% PImax progression, ≥8 weeks duration) are robust to marginal trial additions, though newer devices merit future investigation.

**Action taken: **Search Strategy now states the search window was pre-specified and not extended, with implications detailed in Limitations. 

**Answer 2 (Major Concern 2, PRISMA transparency)**

 We agree completely. The revised PRISMA 2020 flow diagram (Figure 1) now itemizes exclusions at title/abstract (n=58: non-COPD n=19, no IMT n=14, wrong design n=12, no PR n=8, etc.) and full-text stages (n=5: reviews n=3, non-English n=2), fully conforming to PRISMA 2020 standards.

**Action taken:** Figure 1 redrawn with quantified exclusion reasons; Results text updated accordingly. Uploaded to extended data.

**Answer 3 (Major Concern 3, PICOS table)**

 We fully agree. New Table 2 provides PICOS-structured summary for all nine RCTs: population, IMT protocol (device, %PImax, duration, frequency, progression), comparator, outcomes, and key findings with p-values.

**Action taken: **Table 2 inserted. Uploaded to extended data.

**Answer 4 (Major Concern 4, Protocol descriptions)**

 Agreed. Detailed IMT/PR parameters now reside in Results ("Study intervention details" subsection), while Methods retains only eligibility-level PICOS framing.

**Action taken:** Methods Interventions shortened to PICOS eligibility; detailed synthesis (device types, intensities, progression schedules, PR components) moved to new Results subsection post-Study Characteristics.

**Answer 5 (Major Concern 5, Discussion enhancement)**

 We have expanded mechanistically: 

 (i)    inspiratory weakness → dyspnea → hyperinflation chain

 (ii)    why IMT+PR exceeds PR alone via supra-threshold loading

 (iii)    heterogeneity linked to Table 2 protocol parameters.

**Action taken: **Three new/replacement Discussion paragraphs added as detailed previously.

**Answer 6 (Minor Concern 1, Methods/Results boundary)**

 Agreed. Detailed intervention descriptions removed from Methods, replaced by concise PICOS eligibility; full synthesis now in Results.

**Answer 7 (Minor Concern 2, Heterogeneity support)**

 Correct. Table 2, new Results subsection, and rewritten Discussion heterogeneity paragraph now anchor variability to specific protocol rows.

**Also,**

 •    Previous Table 2 ("IMT Effects on Outcome Measures") relabeled as Table 3

 •    Six new references added: Laveneziana et al. (2015); Levine et al. (1997); Ottenheijm et al. (2006); O'Donnell et al. (2009); Rossi et al. (2015); Xie et al. (2025)

**We again thank Dr. Patsaki Irini for her insightful and constructive feedback, which has substantially strengthened the rigor, clarity, and clinical relevance of our work**

# Reviewer report: Reviewer response for version 1

**DOI:** 10.5256/f1000research.193598.r452337

**Published:** 2026-02-25

**Authors:** Noppawan Charususin

**Affiliations:** 1 Thammasat University, Bangkok, Bangkok, Thailand

The authors performed a systematic review to examine whether inspiratory muscle training plus pulmonary rehabilitation provides benefits beyond PR alone in adults with moderate to severe COPD. This systematic review provides moderate-quality evidence that inspiratory muscle training, when incorporated into pulmonary rehabilitation programs, yields consistent improvements in health-related quality of life for individuals with COPD.

 I have two major concerns; the first one is the rational for performing this systematic review

 since the recent Cochrane Database Syst. Rev. (Ammous et al., 2023) has already performed this systematic review as mentioned in the discussion section. What are the strong reasons to support this systematic review again after only a few years? The reason should not be only the different search strategies.

 Secondly, what is the reason why the authors did not register in PROSPERO? Although all methods strictly follow the Cochrane Collaboration standards and PRISMA 2020 guidelines. It would be better to register this review in PROSPERO.

Are the rationale for, and objectives of, the Systematic Review clearly stated?

Partly

Is the statistical analysis and its interpretation appropriate?

Partly

If this is a Living Systematic Review, is the ‘living’ method appropriate and is the search schedule clearly defined and justified? (‘Living Systematic Review’ or a variation of this term should be included in the title.)

Yes

Are sufficient details of the methods and analysis provided to allow replication by others?

Yes

Are the conclusions drawn adequately supported by the results presented in the review?

Yes

Reviewer Expertise:

Pulmonary rehabilitation, inspiratory muscle training

I confirm that I have read this submission and believe that I have an appropriate level of expertise to confirm that it is of an acceptable scientific standard, however I have significant reservations, as outlined above.

# Response

**Published:** 2026-03-03

**Authors:** Fahad Algharbi

**Affiliations:** College of Applied Medical Sci, Imam Abdulrahman bin Faisal University, Dammam, Saudi Arabia

Thank you for your careful review and thoughtful feedback; we greatly appreciate your insights and time.

 Answer 1:

 Thank you for this important point. We agree that the Cochrane review by Ammous et al. is the highest-level synthesis on IMT in COPD, both as a stand-alone intervention and when combined with PR, and we wish to clarify the distinct and complementary rationale for our review. 

 While the Cochrane review evaluates two broad questions together (IMT alone and IMT plus PR) the key operational decision in clinical services is specifically whether adding IMT to an established PR pathway is worth the additional time, equipment, supervision, and adherence burden; our review isolates this integration question entirely, synthesizing outcomes and protocols exclusively in the context where PR is already being delivered, which is the precise scenario clinicians and rehabilitation programs face. 

 Second, the Cochrane authors themselves explicitly acknowledge that a larger effect in people with inspiratory muscle weakness and with longer training durations remains to be confirmed, and that future research should focus on people with weakened breathing muscles; our review was designed to respond to this exact gap by extracting trial-level details that matter for service delivery ( baseline PImax, intensity progression, duration, device type, and supervision ) and by examining whether clinically meaningful signals appear stronger in more impaired patients and longer programs, thereby providing an implementation-oriented synthesis that directly addresses the uncertainty Cochrane identified rather than simply replicating its conclusions. 

 Also, the Ammous et al., 2023 review applied strict methodological exclusions, removing trials that used resistive devices without a controlled breathing pattern or with training loads below 30% of PImax; while methodologically rigorous, this means that many protocols actually used in PR settings ( particularly in frailer patients or early training phases ) are not represented in the Cochrane evidence base, and our broader, PR-embedded protocol mapping provides practical insight into what appears effective, tolerable, and deliverable across the range of real-world rehabilitation pathways. 

 Lastly, and perhaps most importantly, while Cochrane reports that PR plus IMT probably increases PImax without reaching their cited MCID of 17.2 cmH₂O, and that effects on 6MWD and HRQoL versus PR alone are uncertain or small at the pooled level, our review complements this by translating study-level findings into clinically interpretable ranges, demonstrating individual trial PImax gains of 5.2 to 22.9 cmH₂O, identifying that 100% of studies assessing HRQoL across multiple instruments “SF-36, CCQ, ABC, and BBS” reported statistically significant improvements that the SGRQ-focused Cochrane meta-analysis does not capture, highlighting where within-group changes exceed established MCID thresholds for 6MWT and dyspnea, and identifying the ceiling-effect pattern in patients with preserved baseline inspiratory strength that the Cochrane subgroup analysis could not resolve, all of which translate the existing evidence into a format directly applicable to patient selection and clinical decision-making in ways that a pooled mean difference structurally cannot.

 Answer 2:

 The review was registered in PROSPERO (ID: CRD420251251860), and the manuscript has been updated accordingly.
